# Antioxidant and Hepatoprotective Effects of *Croton hypoleucus* Extract in an Induced-Necrosis Model in Rats

**DOI:** 10.3390/molecules24142533

**Published:** 2019-07-11

**Authors:** Thania Alejandra Urrutia-Hernández, Jorge Arturo Santos-López, Juana Benedí, Francisco Jose Sánchez-Muniz, Claudia Velázquez-González, Minarda De la O-Arciniega, Osmar Antonio Jaramillo-Morales, Mirandeli Bautista

**Affiliations:** 1 Área Académica de Farmacia, Universidad Autónoma del Estado de Hidalgo, Mariano Abasolo 600, Colonia Centro, Pachuca, Hidalgo CP 42000, Mexico; 2 Departamento de Farmacología, Farmacognosia y Botánica, Facultad de Farmacia, Universidad Complutense de Madrid, Plaza Ramón y Cajal S/N, 28040 Madrid, Espana; 3 Departamento de Nutrición y Ciencia de los Alimentos, Facultad de Farmacia, Universidad Complutense de Madrid, Plaza Ramón y Cajal S/N, 28040 Madrid, Espana

**Keywords:** antioxidant activity, hepatoprotective effect, *Croton hypoleucus*, oxidative stress

## Abstract

The aim of this study was to evaluate the antioxidant and hepatoprotective activity of *Croton hypoleucus* (EC). The present work reports the first pharmacological, toxicological, and antioxidant studies of EC extract on liver injury. Liver necrosis was induced by thioacetamide (TAA). Five groups were established: *Croton* Extract (EC), thioacetamide (TAA), *Croton* extract with thioacetamide (EC + TAA), vitamin E with thioacetamide (VE + TAA) and the positive control and vehicle (CT). For EC and EC + TAA, Wistar rats (n = 8) were intragastrically pre-administered for 4 days with EC (300 mg/kg.day) and on the last day, EC + TAA received a single dose of TAA (400 mg/kg). At 24 h after damage induction, animals were sacrificed. In vitro activity and gene expression of superoxide dismutase (SOD), catalase (*Cat*), and Nrf2 nuclear factor were measured. The results show that EC has medium antioxidant properties, with an IC_50_ of 0.63 mg/mL and a ferric-reducing power of 279.8 µM/mg. Additionally, EC reduced hepatic damage markers at 24 h after TAA intoxication; also, it increased *SOD* and *Cat* gene expression against TAA by controlling antioxidant defense levels. Our findings demonstrated the hepatoprotective effect of EC by reducing hepatic damage markers and controlling antioxidant defense levels. Further studies are necessary to identify the mechanism of this protection.

## 1. Introduction

Medicinal plants play a key role in the human health care system [1]. According to the World Health Organization, between 65% and 80% of the populations of developing countries currently use medicinal plants [2], as extracts, infusions, or bioactive compounds to treat primary conditions [3,4]. Several pharmacology studies have shown the role of medicinal plants on the treatment and prevention of liver diseases [5]. The biological and hepatoprotective activity of plant extracts defends hepatocytes against lipid peroxidation and other oxidative effects [6] as free radicals, toxic, viral, and bacterial agents [7]. The hepatoprotective activity of plants has been related to several compounds, like flavonoids (isoflavones, anthocyanins, catechins, quercetins), saponins, coumarins, alkaloids, and terpenes [5]. In the complementary traditional medicine, some *Croton* species are highlighted due to their anti-inflammatory, antiseptic, antinociceptive, antiplasmodic, antiproliferative, antiviral, and antibacterial properties, and some compounds, like terpenes, steroids, and flavonoids, have been identified in the *Croton* species. These compounds have important biological activities with therapeutic and medicinal value [3], as anethol from *C. zehtneri* [8]; triterpenes from *C. oblongifolius* [9]; and alkaloids, flavonoids, and glycosides in *C. sparciflorus* [10]. *Croton hypoleucus*, known as Palo blanco and Soliman Liso, is a native shrub of Hidalgo, Mexico. An infusion of its aerial parts is used in treatments of stomachache and pain. In a preliminary phytochemical screening of EC, we found the presence of saponins, alkaloids, tannins, flavonoids, sterols, terpenoids, and carbohydrates as they have been reported before in *Croton* species [3]. To our knowledge, biological studies of *C. hypoleucus* have not been previously reported, but some of its metabolites have been identified in hexane extract, such as three epoxy-clerodane bearing furan rings, named hypoleins A–C and the Crotonpenes A–B [11]. 

Liver is the main metabolic and detoxifying organ that first contacts and neutralizes xenobiotic [12] due to a cellular system of detoxification (cytochrome P_450_, flavin-containing monooxygenase, glutathione transferase), which provides biotransformation of some xenobiotics to toxic intermediates, leading to liver toxic injury [13]. Acute toxic liver injury is characterized by membrane damage, massive necrosis of hepatocytes, infiltration of parenchyma by neutrophils, and activation of hepatic stellate cells, followed by a release of proinflammatory cytokines and the formation of reactive oxygen species (ROS) as the main factors that damage liver cells [14]. ROS are oxygen-containing molecules, including superoxide, hydrogen peroxide, and hydroxyl radical, that are highly reactive with other complex molecules in the cells, such as protein, DNA, and lipids. Endogenous radical scavengers, like antioxidant enzymes, including superoxide dismutase (SOD) and catalase (Cat), can lead to ROS degradation [15]. Nuclear factor erythroid 2-related factor 2 (*Nrf2*) functions as a xenobiotic-activated receptor to regulate the adaptive response to oxidants and electrophiles [16], and the repair and removal of damaged proteins [17]. Activation of *Nrf2* enhances the levels of antioxidant enzymes and phase-2- detoxifying enzymes by complex mechanisms, and this may be one of the ways to reduce oxidative/nitrosative stress and chronic inflammation [18].

Thioacetamide (TAA) is known as a hepatotoxicant, and is used to induce acute and chronic liver injury due to its effects on protein synthesis, RNA, and DNA [19]. TAA hepatotoxicity requires metabolic activation by CYP2E1 with the formation of the reactive metabolites, S-oxide (TASO) and S, S-dioxide (TASO_2_) [20,21]. These active intermediates lead to the formation of adducts of proteins, lipids, and nucleic acids, as well as the formation of ROS, which promote lipid and protein peroxidation and mitochondrial damage [22]. The selective destruction of perivenous hepatocytes and proliferative liver cells allows the TAA model to be used in experimental tests to study the hepatic response against aggressive attack from xenobiotics and to identify the molecular, biochemical, and physiopathological mechanisms though which the hepatic lesion develops [5]. Due to taxonomy characteristics and the pharmacological and chemistry nature of the *Croton* genus, *Croton hypoleucus* could be a source of hepatoprotective compounds. In this sense, the aim of the present work was to evaluate the antioxidant and hepatoprotective effect of EC in thioacetamide-induced liver damage in a rat model. 

## 2. Results

### 2.1. Purification of Main Compounds in Dichloromethane Fraction

**Figure 1:**
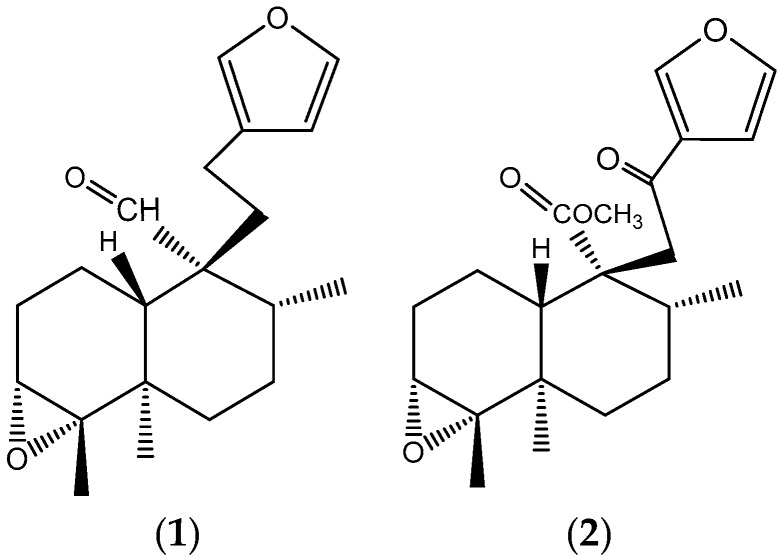
Clerodane-type diterpenoids identified in the dichloromethane fraction of EC: hypolein B (**1**) and Crotonpene B (**2**).

Two clerodane-type diterpenoids were isolated from the dichloromethane fraction of EC. According to ^1^H and ^13^C NMR shifts, they were identified as hypolein B (35 mg) and Crotonpene B (21 mg) (Figure 1). The ^1^H and ^13^C NMR shifts are presented.

Hypolein B: Yellow oil. ^1^H NMR (400 MHz) CDCl_3_: δ 9.92 (s, H-20), 7.96 (s, H-16), 7.37 (dd, J = 1.6, 1.6, H-15), 6.24 (dd, J = 1.6, 0.4, H-14), β2.30, α2.17 (H-12), α1.63 (H-8), α1.66, β1.65 (H-7), α1.77, β1.46 (H-6), α0.95 (H-19), β1.20 (H-18), α1.90, β2.15 (m, H-2), α1.37, β1.53b (m, H-1). ^13^C NMR (100 MHz) CDCl_3_: δ 146.75 (C-16), 138.58 (C-15), 124.72 (C-13), 110.86 (C-14), 60.15 (C-3), 53.27 (C-9), 37.27 (C-5, C-6), 35.29 (C-8), 29.70 (C-11), 27.79 (C-2, C-7), 19.78 (C-18), 17.56 (C-12), 17.45 (C-19), 17.82 (C-17), 15.86 (C-1).

Crotonpene B: Colorless oil. ^1^H NMR (400 MHz) CDCl_3_: δ 7.96 (s, H-16), 7.37 (dd, J = 1.6, 1.6, H-15), 6.24 (dd, J = 1.6, 0.4, H-14), 3.63 (br. S, H-20), α2.07, β2.17, H-2), β1.84 (m, H-10), β1.81 (m, H-8), α1.70 (d, J = 1.2, H-6, H,7), β1.48, α1.58 (dd, J = 2, 1.6, H-1), β1.47 (m, H-6), β1.46 (m, H-7), 1.19 (s, H-18), 1.10 (s, H-17), 0.89 (s, H-19). ^13^C NMR (100 MHz) CDCl_3_: δ 195.21 (C-12), 173.88 (C-20), 146.75 (C-16), 144.03 (C-15). 128.28 (C-13), 108.87 (C-14), 51.43 (C-20), 43.12 (C-11), 38.40 (C-8). 37.27 (C-5, C-6), 27.97 (C-2), 19.78 (C-18), 18.63 (C-1), 17.98 (C-17), 14.11 (C-19). 

### 2.2. Antioxidant Activity of EC

In this study, the tested doses (2 to 6 mg/mL) were antioxidant dose dependent. The inhibition percentages were 28.13% and 78.36% to 2 and 6 mg/mL, respectively. According to the dose-response results, we estimated the IC_50_, as the extract concentration required to reduce the initial concentration of DPPH to 50%. In this case, the EC IC_50_ was 0.6307 mg/mL. To the FRAP assay, EC had an Fe^+3^ ion reducing capacity of 279.8 ± 3.3 µM. Eq. Trolox/mg. 

### 2.3. Acute Toxicity of EC

In the LD_50_ assay, no animals died during the 14 experimental days after administration of 10, 100, and 1000 mg EC/kg in the first phase, and 1600, 2900, and 5000 mg EC/kg in the second phase (Table 1). 

### 2.4. Liver Damage Biomarkers 

**Figure 2:**
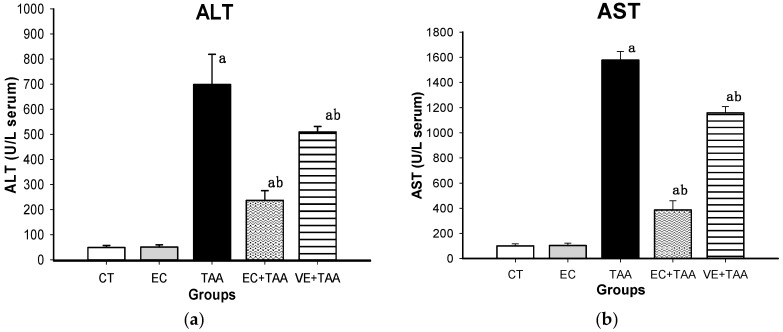
Effect of EC pretreatment on levels of (**A**) ALT and (**B**) AST analyzed by Wiener Lab equipment in the serum of rats intoxicated with a sublethal dose of thioacetamide (TAA). All data are expressed in U/L. Bars indicate the mean value with SE of two determinations (n = 8). The differences compared with the vehicle are expressed as “**a**”; while the differences due to TAA are expressed as “**b**”, *p* ≤ 0.05.

In order to assess the degree of liver injury, biochemical parameters were estimated. The TAA effect on transaminases enzymes is shown in Figure 2. Remarkably, the TAA group presented the highest levels of AST and ALT when they were contrasted with the rest of the treatments. EC and CT did not show significant differences (*p* ≤ 0.05) with each other, as well as VE and EC (data not shown). In the EC + TAA group, ALT and AST presented a decrease of 65.9% and 75.8%, respectively, against TAA, with significant differences between groups. A similar behavior was observed for the positive control (VE + TAA).

**Figure 3:**
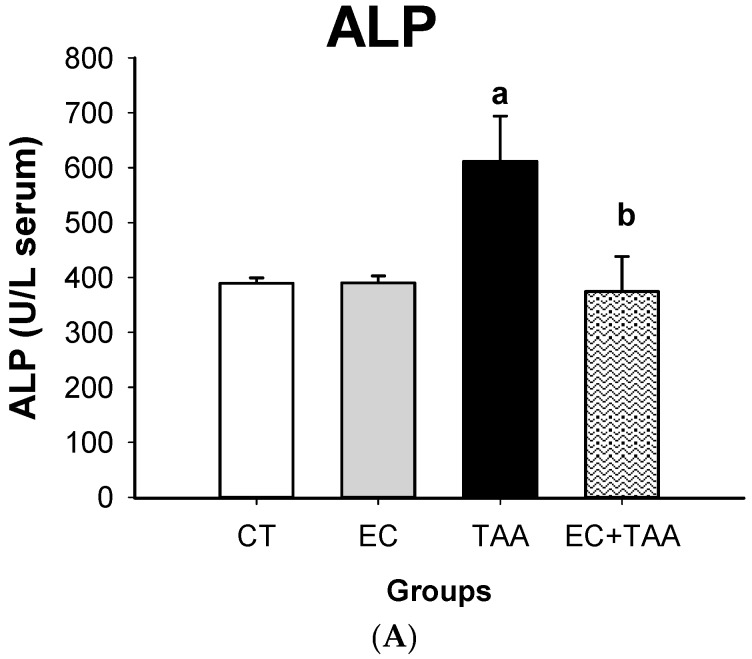

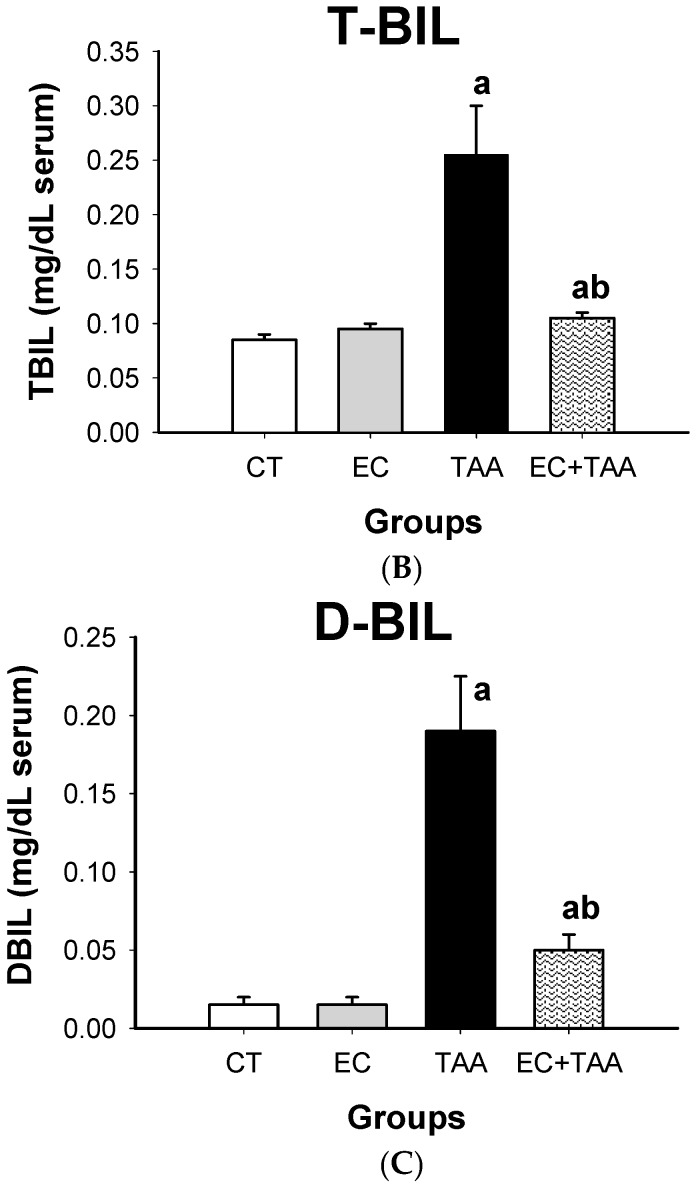
Effect of EC pretreatment on levels of (**A**) ALP, (**B**) T-Bil, and (**C**) D-Bil analyzed by Wiener Lab equipment in the serum of rats intoxicated with a sublethal dose of thioacetamide (TAA). In ALP, the results are expressed in U/L, while T-Bil and D-Bil are expressed in mg/dL of serum. Bars indicate the mean value with the SE of two determinations (n = 8). The differences compared with the vehicle are expressed as “**a**”; while the differences due to TAA are expressed as “**b**”, *p* ≤ 0.05.

**Figure 4:**
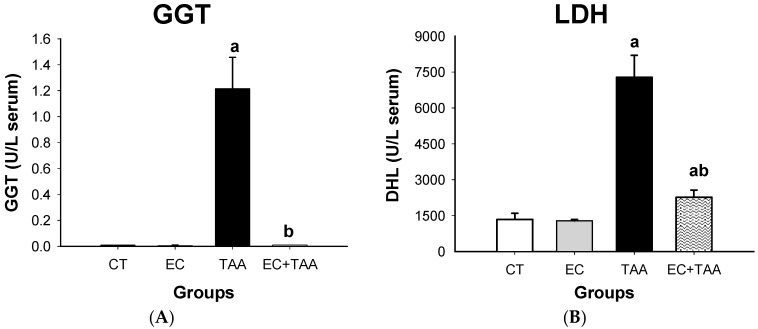
Effect of EC pretreatment on the levels of (**A**) GGT and (**B**) LDH analyzed by Wiener Lab equipment in the serum of rats intoxicated with a sublethal dose of thioacetamide (TAA). The results are expressed in U/L of serum. Bars indicate the mean value with the SE of two determinations (n = 8). The differences compared with the vehicle are expressed as “**a**”; while the differences due to TAA are expressed as “**b**”, *p* ≤ 0.05.

The EC + TAA group showed a significant reduction (38.75%) of ALP levels, which was comparable to the EC and CT groups. A similar tendency was observed in EC + TAA for the T-Bil and D-Bil plasma concentration, which showed a reduction of 58% and 73%, respectively (Figure 3). The TAA groups also presented elevated levels of GGT and LDH (1.21 ± 0.24 × 10^−4^ and 7291 ± 907, respectively). While, EC + TAA exhibited a reduction of 99% and 68.9% to GGT and LDH, respectively (Figure 4).

### 2.5. SOD and Cat Evaluation from Antioxidant Enzyme Activity 

**Figure 5:**
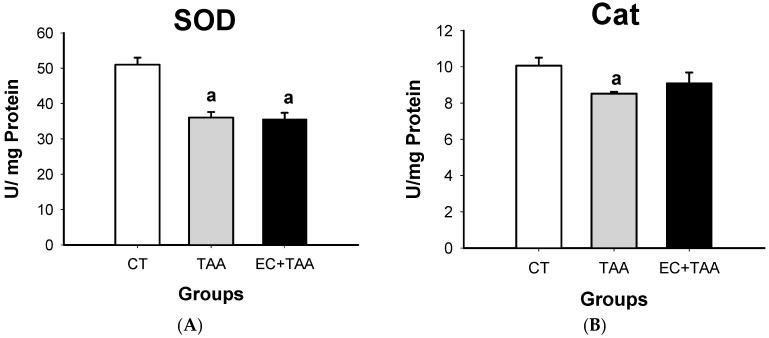
Effect of EC pretreatment on levels of (**A**) SOD and (**B**) Cat enzyme activities in rats’ liver intoxicated by a sublethal dose of thioacetamide (TAA). The results are expressed in U/mg protein. Bars indicate the mean value with the SE of two determinations (n = 8). The differences compared with the vehicle are expressed as “**a**”; while the differences due to TAA are expressed as “**b**”, *p* ≤ 0.05.

To assess the enzymatic antioxidant response of the liver, SOD and Cat activities were measured. The results showed that compared with the vehicle, the levels of both enzymes in EC + TAA (35.58 ± 1.7 and 9.09 ± 0.59 U/mg) decreased at 24 h after TAA administration, as TAA did (36.03 ± 1.56 and 8.52 ± 0.10 U/mg) for SOD and Cat, respectively (Figure 5). For Cat, no significant differences (*p* ≤ 0.05) were reported between CT and EC + TAA.

### 2.6. MnSOD, CuZnSOD, Cat, and Nrf2 mRNA Expression

**Figure 6:**
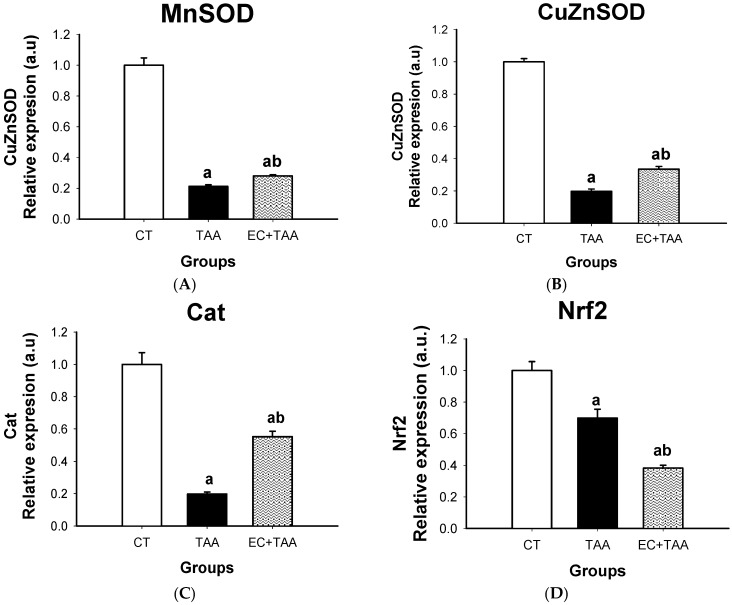
Effect of EC on (**A**) MnSOD, (**B**) CuZnSOD, (**C**) Cat, and (**D**) *Nrf2* expression in homogenated liver of rats intoxicated with a sublethal dose of thioacetamide (TAA). The results are expressed in relative expression, arbitrary units (a.u.). Bars indicate the mean value with the SE of two determinations (n = 8). The differences compared with the vehicle are expressed as “**a**”; while the differences due to TAA are expressed as “**b**”, *p* ≤ 0.05.

Results showed that TAA treatment led to a significant reduction of *MnSOD*, *CuZnSOD*, *Cat*, and *Nrf2* gene expression (Figure 6). On the other hand, the EC + TAA group stimulated relative mRNA expression of MnSOD, CuZnSOD, and Cat in comparison with its TAA counterparts, but without reaching similar values to the CT animals. In contrast, the *Nrf2* expression displayed a significant reduction in the EC + TAA group in comparison with both the CT and TAA groups.

## 3. Discussion

### 3.1. Main Compounds in Dichloromethane Fraction

Diterpenoids are characteristic components of the *Croton* species [3]. They represent 85% of terpenoid compounds identified in these species [23]. Apart from the Clerodane-type terpenoid, hypolein B and Crotonpene B have also been reported for *Croton hypoleucus* by Velazquez et al. [11] in the hexane extract of the aerial parts, as well as by Sun et al. [24] in the methanol extract of aerial parts of *Croton yanhuii.* Diterpenoids are well known as compounds with remarkable biological activities, such as anti-tumor, anti-malarial, anti-inflammatory, antimicrobial [25], hepatoprotective [26,27,28,29], and cytotoxic [30]. Some diterpenes may be toxic for humans, resulting in acute or chronic impacts on different tissues and organs. Kubo et al. [31] showed that the *nor*-diterpene *trans*dehydrocrotonin isolated from *C. cajucara* is responsible for its hepatotoxicity, while the extracts of the same plant showed hypolipidemic activity and an absence of hepatotoxicity in animal models [32]. Also, toxicity occurs when it is used long term or is taken at high doses [33]. 

### 3.2. Antioxidant Activity of EC

The in vitro antioxidant activity of the crude extract was evaluated through 2,2-diphenyl-1-picrylhydrazyl (DPPH) and the ferric reducing ability of plasma (FRAP) assays. The DPPH molecule can accept an electron from a hydrogen radical to become itself a stable molecule, and then it reacts with a reducing agent to form a new bind [21], so the DPPH assay determinates the EC capacity to scavenge free radicals as an antioxidant power measurement [34]. The percent of inhibition reported for EC is comparable with the ethanolic extract of *C. zambesicus*, which presented an inhibition of 72% [35]. On the other hand, IC_50_ is a widely used parameter to measure the antioxidant activity of extracts [36]. *Croton* species leave extracts of *C. argyrophyllus* and *C. heliotropiifolius* showed IC_50_ values of 0.22 and 0.352 mg/mL, respectively [34], while *C. leptstachyus* and *C. bonplandianum* have IC_50_ values of 11.6 and 416 µg/mL, respectively. The IC_50_ was 0.6307 mg/mL, which indicates that a higher EC concentration was needed to scavenge 50% of the DPPH free radical, as an antioxidant potential [37].

The FRAP assay is based on the reduction, at low pH, of a colorless ferric complex (Fe^3+^-tripyridyltriazine) to a blue ferric complex (Fe^2+^-tripiridyl-*s*-triazine) by the action of electron donating antioxidants [38]. The reduction is monitored by measuring the change in absorbance at 593 nm [39]. The extract composition is preponderant for the and the extract is rich in flavonoid-type phenolic compounds. Flavonoid compounds, such as quercetin, kaempferol, quercitrin, and 3-O-methyl ether [40], have been recognized in the *Croton* genus [3]. The participation and synergisms of flavonoid compounds could contribute to the EC antioxidant capacity [41], and their interactions with other compounds could potentiate or interfere with the EC antioxidant ability [42]. The antioxidant and inhibition of free radical production are important for the protection of cells from TAA-induced hepatotoxicity [43]. Different mechanisms, in which antioxidant compounds perform their scavenging properties, have been documented: They act as a physical barrier to prevent ROS generation, and they could access target biological sites, as a chemistry trap catching energy and chelating electrons; as a reactive species scavenging catalytic system and breaking redox chains, and scavenging radicals; or binding to targeted metal compounds and avoiding redox chain formation [44]. Most of them depend on the hydrogen atom transference rate from the compounds to the radicals [45]. The results of this experiment showed that EC may contain potential compounds able to donate hydrogen atoms to free radicals to become more stable molecules, and are responsible for the reported antioxidant activity. The EC capacity to the scavenging DPPH radical and reducing Fe^3+^ can contribute by reducing oxidative stress effects and liver damage. The discovery of antioxidant compounds is critical for new drug research and the treatment of diseases related to oxidative stress.

### 3.3. Acute Toxicity of EC

Acute toxicity testing is the defining and evaluating of a toxic syndrome produced by a single dose or a few doses of an extract or drug administered over the course of a day [46]. During the observation period, rats breathed, ate, and increased body weight normally. Conditions, such as difficulty to breath, loss of appetite, and death, are signs of toxicity [47]. During the post mortem examination, the macroscopic morphology of the liver, spleen, lungs, kidneys, and stomach showed normal color and morphology (data not showed) compared with the vehicle. A lot of *Euphorbiaceae* species are known in different countries as being toxic or medicinal plants. Given its therapeutic response, its chemical diversity can be hypothesized. Compounds, like alkaloids and forbol esters, have been reported in *Croton* species [3]. Different extracts and essential oils of *Croton* species, such as *C. membranaceus*, *C. sparsiflorus, C. bonplandianum*, and *C. zehntneri*, have been evaluated to find their toxicity level at different doses from 300 to 5000 mg/kg [10,48,49] and are considered safe. Nevertheless, a dose of 447.18 mg/kg of *C. polyandrus* essential oil produced the death of mice [50]. The extract toxicity depends on several factors, such as the chemistry composition, doses, and exposition time. In our study, the rat’s survival in all evaluated doses during the two study weeks, and a higher 5000 mg/kg suggests that ethanolic *C. hypoleucus* extract is not toxic [51].

### 3.4. Liver Damage Biomarkers 

The liver damage induced by xenobiotic agents as TAA is characterized by an increase of serum liver enzymes. TAA is a toxic agent that causes hepatocytes necrosis and it contributes to cirrhosis development through multiple action mechanisms, such as oxidative stress, decrease of the antioxidant system response, and lipid peroxidation [52]. Particularly, AST and ALT transaminases are used as biomarkers of hepatocellular necrosis. The serum transaminases concentration is referred as an indicator of the liver damage severity [53,54,55,56]. ALT is present in the liver at higher concentrations than other organs. AST is considered to have a lower specificity for liver damage than ALT due to it is presence in other organs [57]. Along with TAA metabolism, thioacetamide-s-oxide and reactive species are produced. The reactive species harm the cell by lipid peroxidation and produce a breakdown and loss of permeability of the cellular membrane [54]; this is a probable explanation of the increase in AST and ALT levels from the TAA group. These results are in accordance with several studies on TAA-induced liver necrosis in experimental animals [58,59,60,61]. The present results also demonstrated the protective role of vitamin E against TAA, and they were in line with a preliminary study [62]. EC + TAA showed a significant decrease in the serum levels of ALT and AST in relation with VE + TAA and TAA (*p* ≤ 0.05). The results in EC + TAA are strongly related to the EC capacity to save the cell against necrotic damage produced by TAA to reduce the rate of transaminase release and cellular membrane stabilization [44]. 

ALP is a hydrolase enzyme, which is eliminated by bile. It is present in cells covering biliary conducts, as well as other organs, like bone, placenta, kidney, and intestine. Hepatotoxicity leads to an elevation of normal values due to the body´s excretion inability through bile due to the congestion or obstruction of the biliary tract, which may occur within the liver, such as was observed in the TAA group. The result showed for the EC + TAA indices that EC has the ability to reduce the effects of bile obstruction induced by TAA by decreasing ALP toward vehicle levels (Figure 3). A similar behavior was reported in bilirubin determination. The bilirubin is a product from regular hemoglobin breakdown, and it is released into the bile [54]. The T-Bil and D-Bil result to EC + TAA indicates that EC contributed to bilirubin metabolism after induced damage by TAA. 

For D-Bil and T-Bil plasma concentrations, TAA lead to elevated levels of bilirubin. The induced liver damage by TAA caused the liver to lose its ability to conjugate to bilirubin; thereby, its excretions are affected, and it causes hyperbilirubinemia in serum. This alteration, along with higher transaminases levels, is a sign of acute or toxic injury [61] as the TAA group showed. GGT enzyme is localized in the liver, kidney, and pancreas. It catalyzes the conjugation of electrophilic species from TAA metabolism with GSH [53]. GGT levels tends to increase due to its release from the hepatocytes to the circulatory system by changing the membrane permeability. Although the mechanisms for GGT induction are uncertain, they have been associated with C-reactive protein, a general marker for increased oxidative stress, which leads to overconsumption of GSH with a compensatory increase in GGT synthesis [62,63].

On the other hand, GGT reflects a state of oxidative stress forward to chronic disease; while LDH increases its levels as a result of liver diseases [64]. The liver biomarker results describe the protective ability of EC against free radicals and electrophilic compounds from TAA biotransformation, which promotes cellular stability, serum transaminases and bilirubin depuration, as well as recovery competence, thus keeping biomarker levels closer to the vehicle, as *C. oblongifolius* ethanol extract [9], *C. zehnteneri* essential oil [8], *C. sparciflorus* [10], and *C. bonplandianus* methanol extract [21] have shown against necrotic effects of CCl_4_, acetaminophen, and N-nitrosodietylamine. The liver biomarkers’ regulation represents the liver’s recovery to a normal state [65]. To date, the compounds responsible for the hepatoprotective activity of *C. hypoleucus* have not been revealed, however, several studies [8,9,10] report that flavones, terpenoids, alkaloids, tannins, and saponin may be responsible for this pharmacological effect.

### 3.5. SOD and Cat System

Defensive responses of organisms to oxidative stress include the utilization of endogenous antioxidant enzyme systems, lipid soluble and water-soluble antioxidant molecules, and phytochemicals, which can be detected through measurement of the total antioxidant capacity. Antioxidants, such as SOD, Cat enzymes, and GSH, are some of the most important elements that act as a defense against oxidative damage. They keep ROS at low levels and avoid excessive production [66]. For the purpose of this investigation, the effect of EC was evaluated on the levels of SOD and Cat as enzymes of the system of antioxidant defense. SOD is present in the cytoplasm and mitochondria of cells. The SOD molecule in the cytoplasm contains copper and zinc atoms, while mitochondrial SOD contains manganese. SOD catalyzes superoxide radical dismutation (·O_2_) into hydrogen peroxide (H_2_O_2_); even though H_2_O_2_ is not a radical, it is rapidly converted into hydroxyl radical, which is highly reactive, by means of the Fenton reaction and Cat enzymatic activity [67]. The regulatory activity of this enzyme enables mutual protection; when the superoxide radical is produced, it is disabled by Cat, while H_2_O_2_ inhibits SOD [68]. Hepatotoxicity by TAA requires metabolic activation with the formation of the reactive metabolites, S-oxide (TASO) and S, S-dioxide (TASO_2_) [21], which bind to microsomal lipids, leading to is peroxidation, as well as ROS production, such as hydroxyl, peroxide, and superoxide radicals. ROS affect antioxidant defense mechanisms, and they decrease SOD, Cat, and GPx activity, leading to liver damage, cirrhosis, and hepatocellular carcinoma [54]. In our study, the acute liver injury by TAA was characterized by a reduction in the in vitro activity of SOD and Cat (Figure 5) due to the attack of superoxide and hydrogen peroxide radicals against the cell [68]. The TAA administration to rats may cause cellular structure changes, interfere with RNA movement from nuclei to the cytoplasm, and reduce the number of viable hepatocytes, as well as reduce the oxygen intake rate. TAA prolonged exposure leads to hyperplastic nodule formation, hepatocellular carcinoma, and cirrhosis. The induced cirrhosis by TAA in rats has been shown to be an experimental model of disease comparable with human ethology and pathology [69]. Higher Cat levels compared to SOD means that EC could promote the antioxidant defense system by increasing Cat activity against H_2_O_2_ and protecting cells against acute toxic liver damage at 24 h after TAA administration, as shown in Figure 5 shows.

### 3.6. MnSOD, CuZnSOD, Cat, and Nrf2 mRNA Gene Expression

Gene expression is a process by which DNA instructions are converted into functional products as proteins. In *MnSOD, CuZnSOD*, and *Cat*, the lower gene expression was derived from the process of TAA detoxification, which produces an attack of hydroxyl radicals and DNA damage. The results highlight a greater *Cat* gene expression due to EC + TAA toward basal stages, which means that EC has an inhibitory capacity against ·O_2_ and H_2_O_2_, avoiding peroxidation and DNA damage by activation of the defense antioxidant system [21]. These results are the opposite to the *Nrf2* gene expression, which was suppressed. *Nrf2* plays an important role in the activation of antioxidant enzymes by regulating their transcription. It is primarily regulated by *Keap1* (Kelch-like erythroid cell-derived protein with CNC homology-associated protein 1) dependent ubiquitination-proteasomal degradation and is activated by oxidants [70], so *Keap1* binds *Nrf2* in the cytoplasm and maintains *Nrf2* at a low steady state level [17]. Another mechanism for *Nrf2* degradation is phosphorylation by glycogen synthase kinase 3 (GSK3) via β-transducin repeats-containing protein (β-TrCP)-Cul1-based ubiquitin ligase [71]. As a consequence, *Nrf2* knockout means that the effect could also be mediated by inflammatory cells [68]. Although *Nrf2* activation is generally considered to have a beneficial effect in liver disease [72], we found that the antioxidant mechanism inducted by EC was independent of activation of *Nrf2* expression. The increase of levels of antioxidant enzymes by activating *Nrf2* may not be enough to decrease oxidative stress and chronic inflammation optimally due to antioxidants, which tend to decrease in an oxidative environment, and must also be elevated. Besides, the levels of antioxidant can be increased by supplementation or an *Nrf2* independent mechanism [18] as happened with the EC + TAA group.

## 4. Materials and Methods 

### 4.1. Chemicals and Reagents

The main chemicals used in this study include: CDCl_3_, 2,2-Diphenyl-1-picrylhydrazyl, thioacetamide, tween 80, 2,4,6-Tris(2-pyridyl)-s-triazine, TPTZ (2,4,6-tri (2-pyridyl-s-triazine) 6-hydroxy-2,5,7,8-tetramethil-chromal-2-carboxylic acid- Trolox, Chloride ferric, Tri-Reagent, and agarose were purchased from Sigma Chemical Co. (St. Louis, MO, USA); pentobarbital was provided by Pisa (Mexico City, Mexico). qPCR Master Mix (Nzytech, Portugal); SYBR Green (Biotools, Madrid, Spain); Silica gel 60, and other chemicals, such as ethanol, methanol, and hydrochloric acid, were reactive grade products from Merck (Darmstadt, Germany). DNase I RNase-free reagents were bought Thermo Fisher Scientific (Waltham, MA, USA).

### 4.2. Preparation of EC

Aerial parts of *C. hypoleucus* were collected on January 2016, from San Vidal, Tulancingo, Hidalgo State [20.116002, −98.305734]. The plant was identified by Manuel González Ledezma, taxonomist of Department of Botany, Autonomous University of Hidalgo State. The specimen, voucher number: DVM01, was deposited at the Herbarium of Biological Sciences Research Center. Aerial parts were dried for a period of 15 days in a light protected area and milled with a commercial grinder. Powdered plant (500 g) was extracted by maceration with 4 L of ethanol for a week in triplicate. The material was filtered and concentrated by a rotatory evaporator (Büchi, Switzerland) at 40 °C. The crude extract obtained was used for in vitro and in vivo analysis.

### 4.3. Purification of Main Compounds in Dichloromethane Fraction

A sample of 5 g of crude extract was fractionated through a flash column chromatography performed over silica gel 60 (230–400 mesh). The sample was eluted with 500 mL of hexane, dichloromethane, ethyl acetate, and methanol to yield 0.3, 1.8, 2.1, and 0.8 g, respectively. The dichloromethane fraction was dried and supported over silica gel 60 column chromatography and eluted with a hexane: Dichloromethane (6:4) mixture. In total, 64 fractions of 10 mL were collected and monitored by TLC on precoated silica gel aluminum sheets. The compounds were visualized through UV detection and by spraying with vanillin/H_2_SO_4_/EtOH solution, followed by heating. The fractions, 48–52, was selected and supported over a preparative layer chromatography with hexane: Dichlorometane (1:1) which lead to the isolation of two compounds (Figure 1), which were characterized by ^1^H and ^13^C NMR spectroscopy using a Spectrometer 400 MHz (Bruker Avance III, Billerica, MA, USA) and CDCl_3_ as solvent. The NMR shifts were compared with literature data [11,24]. 

### 4.4. Antioxidant Capacity of EC

DPPH Free Radical Scavenging Activity. The DPPH radical scavenging was determined according to the method of Brand-Williams [73] with slight modifications. From a 50 mg/mL methanol solution of EC, 50 µL was mixed with 200 µL DPPH reactant (200 µM). The mix of 1 mL methanol and 1 mL DPPH solution was used as the control. The reaction was carried out in triplicate. The mixture was kept for 30 min in a 96-well plate. After incubation, the absorbance was measured at 517 nm using an absorbance plate reader (Fluostar Optima, BMG Labtech, Ortenberg, Germany). The percent of inhibition was calculated by the following equation:

(1) $$\%\;\mathrm{inhibition}=\frac{\mathrm{Abs}\;\mathrm{Control}-\mathrm{Abs}\;\mathrm{CE}}{\mathrm{Abs}\;\mathrm{Control}}\times 100.$$

The IC_50_ or effective concentration values, representing the amount of extract required to decrease the absorbance of DPPH by 50%, were calculated from the percentage of radical scavenging activity.

Ferric-reducing power FRAP. The ferric-reducing power of the crude extract was determined according to a modified protocol of Benzie and Strain [74]. The working FRAP solution was prepared daily by mixing 25 mL of acetate buffer (0.3M pH 3.6), 2.5 mL of 10 mM TPTZ in HCl (40 µM), and 2.5 mL of ferric chloride in distilled water. The working solution was kept at 35 °C and in the dark. In the test, 30 µL of crude extract in methanol (10 mg/mL) was mixed in 90 µL of distilled water plus 900 µL of FRAP solution. The reaction mixture was incubated for 30 min at 37 °C. The absorbance was measured at 595 nm with a spectrophotometer (UVikon 930 spectrophotometer, Kontron Instruments S.A., Madrid, Spain). For the ferric-reducing power determination, ferrous sulfate heptahydrate solution (200–1000 µM) instead of extract was used as the calibration curve. The results (in triplicate) are expressed as µM Fe/mg crude extract.

### 4.5. Experimental Animals

Male Wistar rats weighing 180 to 200 g and aged 7 weeks were used in this study. The animals were obtained from the vivarium of the Autonomous University of Hidalgo State. They were adapted according to appropriate protocols prior to commencement of the experiment. The rats were maintained in clean polypropylene cages in a temperature controlled room and 12:12 h light/dark cycles with ad libitum access to pellet food and water. After 1 week of acclimation, rats were randomly assigned to experimental groups. Besides, all the experiments were conducted by approbation of the Internal and Ethical Committee for the care and use of experimental animals with the official certificate No. 5-12-2017 and according to the Official Mexican Norm (NOM 062-ZOO-1999) [75].

### 4.6. Acute Toxicity

The acute toxicity was evaluated using the Dietrich Lorke assay [52]. This study was conducted in two phases. In the first phase, three groups (n = 3) of rats were administered intragastrically with a homogeneous solution of EC in 1% tween 80 at the respective oral doses of 10, 100, and 1000 mg/kg body weight. The animals were observed frequently for 14 days and any adverse effects (mortality, body weight, water and food intake) were recorded for the 14 days. In the second phase, three new groups (n = 3) rats were administered respective oral doses of 1600, 2900, and 5000 mg/kg body weight of crude extract. In both phases, a vehicle was fed 1 mL of Tween 80.1% intragastrically. The possible number of deaths was recorded and the LD_50_ value was determined. 

### 4.7. Thioacetamide-Induced Hepatotoxicity

The thioacetamide-induced damage was performed following a pre-established protocol [56]. Male Wistar rats were randomly distributed into 5 groups (n = 8). Groups EC and EC + TAA received a single dose intragastrically (i.g.) of crude extract (300 mg/kg body weight) every 24 h for four days of treatment. At the same time, VE + TAA was administered with 100 mg/kg (i.g.) and groups Thioacetamide (TAA) and vehicle (CT) were administered i.g. with a tween 80 solution (1% v/v). On the fourth day, group TAA, EC + TAA, and VE + TAA were administered one dose (400 mg/kg body weight) of TAA dissolved in 1 mL NaCl (0.9%) intraperitoneally (i.p.). Then, 24 h after TAA administration, all animals were sacrificed with intramuscular pentobarbital doses (50 mg/kg body weight) via the i.p. route. Then, through an abdominal dissection, samples of blood were obtained by portal vein puncture. Serum from blood was separated by centrifugation at 4000 rpm, 10 °C, and 15 min and analyzed for various biochemical parameters related to liver damage using well-established protocols. On the other hand, livers were rapidly dissected out and washed using 0.9% NaCl sterile solution and then immediately stored in a −80 °C freezer until tissue homogenate preparation. 

#### 4.7.1. Biochemical Parameters

Quantitative determination of the ALT, AST, ALP, GGT, and DHL enzymes and DB was carried out with WIENER-Lab optimized equipment (Rosario, Argentina). Quantitative determination of ALT enzyme was measured by diminution in absorbance to 340 nm at 25 °C, produced by the oxidation of NADH into NAD^+^ while pyruvate reduction into lactate was done by lactate dehydrogenase [76]. The activity of AST was measured by the diminution in absorbance at 340 nm and 25 °C produced by NADH oxidation to NAD^+^ in the paired reaction of the oxaloacetate reduction into malate by malate dehydrogenase activity [77]. ALP determination was done through pnitrophenylphosphate (pNPP) hydrolysis yielding phosphate and p-nitrophenol at alkaline pH and 405 nm. The *p*-nitrophenolate production is directly proportional to the enzymatic activity of the sample [78]. GGT was analyzed by the change in absorbance at 410 and 480 nm due to the formation of 5-amino-2-nitrobenzoate, a product from a glutamyl group transfer from the donor substrate, gamma-glutamyl-3-carboxy-4-nitroanilide, to the acceptor, glycylglycine, by GGT [79]. LDH catalytic activity was determined by measuring the NAD^+^ formation rate from NADH oxidation in an alkaline pH at 340 nm and 37 °C [80]. The evaluation of bilirubin was performed through production of azobilirubin from the bilirubin reaction with diazotized sulfanilic acid photocolorimetrically measured at 530 nm [81].

#### 4.7.2. SOD and Cat in Vitro Activity

Homogeneous samples of liver tissue were used for the SOD and Cat biochemical assays. Liver tissue was homogenized in phosphate-EDTA buffer (0.1 M sodium phosphate and 0.005 M EDTA, pH 8) at 100 mg/mL, with the addition of 10 μL/mL of perchloric acid. It was then centrifuged at 10,000 rpm for 10 min at 4 °C. Superoxide dismutase (SOD) activity was measured using the Nitroblue Tetrazolium reagent (NBT) method according to Neha and Mishra protocol [82] with modifications. This method is based on the generation of superoxide radical (O^2−^) by autoxidation of hydroxylamine hydrochloride in the presence of NBT, which gets reduced to nitrite. Nitrite in the presence of EDTA gives a color measured at 560 nm. Cat activity was measured as described by the Aebi [83] method using hydrogen peroxide as a substrate. The decomposition of H_2_O_2_ was followed directly by a decrease in absorbance at 260nm. Enzyme activity was standardized to liver homogenate protein concentrations determined according to Bradford´s method [84]. Final enzyme activity results are expressed as IU/mg protein.

#### 4.7.3. Extraction and Quantification of RNA by RT-PCR

RNA samples were isolated from 100 mg of liver using TRI-Reagent and treated with DNase I RNase-free reagents to avoid any contamination with genomic DNA. The yield and quality of RNA was assessed by measuring absorbance at 260, 280, and 310 nm and by electrophoresis on agarose gels (1%). Total RNA of sample was reverse-transcribed to first-strand complementary DNA (cDNA) using Nzytech qPCR Master Mixes. Relative Mn-SOD, CuZn-SOD, Cat, and Nrf2 mRNA levels were quantified with a LightCycler Real-Time PCR Detection System (Roche Diagnostics, Indianapolis, IN, USA), using SYBR Green as the fluorescent binding dye. Detection was monitored by measuring the increase in fluorescence throughout the cycles. The standardization was carried out to the β-actine value. The results are expressed as fold changes of the threshold cycle (Ct) value relative to the vehicle using the 2^−ΔΔCt^ method [85]. The PCR protocol was: Preincubation at 95 °C for 10 min followed by 45 cycles of denaturation at 95 °C for 10 s with an annealing temperature of 60 °C for each couple primer, extension at 72 °C for 15 s, and cooling at 40 °C for 30 s. Primer sequences were as indicated in Table 2.

### 4.8. Statistical Analysis

Significant differences between the results were calculated by variance analysis (ANOVA). One-way ANOVA was determined by Statgraphics centurion XVII.II version (Statistical graphics Corporation, Inc., Rockville, MD, USA). A post-hoc Tukey test was performed to identify significant differences (*p* ≤ 0.05) between treatments. 

## 5. Conclusions

The current study reports the first antioxidant and hepatoprotective evaluation of ethanolic crude extract of *Croton hypoleucus* (EC) in a frame of a thioacetamide-induced (TAA) liver damage model in rats. The main mechanisms by which EC protects the liver from toxic damage are associated with its antioxidant properties and its ability to modulate Cat involved in the antioxidant defense system. Additionally, EC has the ability to recover cell mitochondria and regulate biomarkers of the liver after TAA injury, thus preventing the development of hepatotoxicity. Nrf2-no dependent catalase activation revealed the role of antioxidant mechanisms while biochemical parameters were the first sign of its hepatoprotective activity. *Croton hypoleucus* could offer a novel alternative to the limited therapeutic options that exist for the treatment of liver diseases.

## Figures and Tables

**Table 1:** LD_50_ of EC.

EC	Intragastric Doses (mg/kg)		
Phase I	10	100	1000
Mortality	0/3	0/3	0/3
Phase II	1600	2900	5000
Mortality	0/3	0/3	0/3
LD_50_	>5000		

**Table 2:** Primer sequences used in RT-PCR.

**Mn-SOD**	sense: 50-ACTGAAGTTCAATGGCGGG-30 and antisense: 50-TCCAGCAACTCTCCTTTGGG-30
**CuZn-SOD**	sense: 50-CTTCGAGCAGAAGGCAAGCG-30and antisense: 50-GACATGGAACCCATGCTCGC-30
**Cat**	sense: 50-ATCAG**GGA**TGCCATGTTGTT-30 and antisense: 50-GGGTCCTTCAGGTGAGTTTG-30
**Nrf2**	sense: 50-TTGTAGATGACCATGAGTCGC-30 and antisense: 50-GAGCTATCGAGTGACTGAGCC-30
